# Specificity of anti-SSB as a diagnostic marker for the classification of systemic lupus erythematosus

**DOI:** 10.3892/etm.2013.1051

**Published:** 2013-04-08

**Authors:** LI RAO, GUIYAN LIU, CHUNFEN LI, YAJUAN LI, ZHIGUO WANG, ZIJIAO ZHOU, SHENGQUAN TONG, XIANMING WU

**Affiliations:** 1 Department of Rheumatology, Worker’s Hospital of Tangshan, Tangshan, Hebei 063000;; 2 Department of Rheumatology and Immunology, The Third People’s Hospital of Zhengzhou, Zhengzhou, Henan 450000, P.R. China

**Keywords:** anti-Sjögren’s syndrome type B antibody, linear immunoblot assay, systemic lupus erythematosus

## Abstract

The aim of the present study was to investigate the sensitivity and specificity of anti-Sjögren’s syndrome type B (SSB) antibodies for diagnosing systemic lupus erythematosus (SLE) and to understand the correlation between anti-SSB antibodies and the clinical manifestations of SLE. A line immunoassay (LIA) was used to detect the presence of serum anti-SSB antibodies in SLE patients. The clinical manifestations of the patients were recorded to enable their correlation with the serum anti-SSB antibodies to be analyzed. In 25.7% of the 74 SLE patients, the serum was positive for anti-SSB antibodies, whereas only 3.3% of the 30 control cases were positive. The specificity of anti-SSB antibodies for detecting SLE was 96.7%. In anti-SSB antibody-positive SLE patients, the incidence of cheek erythema, alopecia, serositis, secondary Sjögren’s syndrome (sSS), leukocytopenia, elevated immunoglobulin (Ig)G and positive presence of anti-Sjögren’s syndrome type A (SSA)60 or anti-SSA52 antibodies was higher than in the anti-SSB antibody-negative group (P<0.05). Anti-SSB antibodies are important for the diagnosis of SLE and are associated with cheek erythema, alopecia, serositis, sSS, leukocytopenia, the elevation of IgG and positive presence of anti-SSA60 or anti-SSA52 antibodies.

## Introduction

Systemic lupus erythematosus (SLE) is a multiple system and organ repeated autoimmune disease characterized by a complex, diverse and prolonged course. The pathological overactivation of T and B cells ultimately leads to an abnormal response from the immune system. Autoantibodies are generated which attack the body’s own healthy cells, tissues and/or organs, causing inflammation and damage. For certain autoimmune diseases, there may be an extremely long latency period between the disease onset and clinical presentation. Autoantibodies play a significant role in the early diagnosis and treatment of autoimmune diseases. Currently, anti-double stranded (ds)DNA antibodies and anti-Sm antibodies are used as serum markers to diagnose SLE (1), but they are not conducive to an early diagnosis due to their high specificity and low sensitivity (2,3). Previous studies identified a number of new diagnostically significant autoantibodies, including anti-SmD1 antibodies, in patients with SLE, using ELISA or western blot assays (4–6). A variety of poly ADP-ribose polymerase (PARP) proteomics were identified by Jeoung *et al*(7) using ELISA, and globally, scholars detected that anti-cmDNA antibodies were highly expressed in the serum of SLE patients and provided a higher diagnostic sensitivity and specificity (8–10). An in-depth study of these new autoantibodies is likely to be favorable for the diagnosis and treatment of SLE, but also requires large-scale clinical validation. In fact, clinically, early SLE is prone to misdiagnosis and missed diagnosis, as detection with these antibodies is not yet universal in clinical trials. Anti-Sjögren’s syndrome type B (SSB) antibodies are one of the most common autoantibodies in the serum of SLE patients and also one of the earlier autoantibodies to be produced (11–13). A previous study showed that the anti-SSB antibodies were produced prior to the SLE symptoms appearing (14); on average 3.61±0.38 years earlier than the diagnosis was established and 2.83±0.43 years earlier than the appearance of any clinical symptoms. The anti-SSB antibody-positive rate was 34% prior to the symptom onset, 34% prior to the diagnosis and 35% subsequent to the diagnosis of SLE. Anti-SSB antibodies are significant participants in the pathogenesis of the SLE process and the linear immunity (line immunoassy, LIA) method, which may be used to detect the anti-SSB antibodies of patients with SLE, is simple and reasonably priced. In the present study, the LIA method was used to detect the anti-SSB antibodies of patients with SLE and the clinical significance of an examination to avoid the missed diagnosis of SLE and delayed treatment was discussed.

## Patients and methods

### Patients

A total of 74 patients who had been diagnosed with SLE for the first time were selected from the outpatients and inpatients of the Worker’s Hospital of Tangshan (Hebei, China) between January and October in 2011. The diagnosis was in compliance with the 1997 American Rheumatism Association (ARA) SLE classification diagnostic criteria and excluded infectious or malignant diseases (15). In the same period, 30 patients with rheumatic diseases, including 14 with ankylosing spondylitis, 6 with polymyositis, 3 with dermatomyositis, 3 with osteoarthritis, 2 with Still’s disease and 2 with scleroderma were enrolled as controls. The present study was conducted in accordance with the Declaration of Helsinki and with approval from the Ethics Committee of the Worker’s Hospital of Tangshan. Written informed consent was obtained from all participants. Of these patients, 3 were male and 71 were female. The mean age was 33.16±14.73 years (range, 12–78 years). In the control group 2 cases were male and 28 were female. The mean age was 32.89±15.37 years (range, 11–80 years). There was no statistically significant difference in gender or age between the two groups.

### Serum anti-SSB antibody measurement

The Anti-SSB Antibody Detection kit was purchased from Imtec Immundiagnostika GmbH (Berlin, Germany) and the experiments were conducted in accordance with the manufacturer’s instructions. The key steps involved were the incubation of the reaction strips in the detection chamber with a 1:100 dilution of patient serum for 1 h prior to washing them 3 times; then the addition of 1 ml anti-human immunoglobulin (Ig)G to the reaction strips at room temperature for 30 min and washing them again 3 times; and finally the addition of 1 ml chromogenic substrate agent at room temperature for 10 min, prior to rinsing once, followed by the addition of 1 ml stopping solution. Subsequent to desiccating the strips on the filter paper, the results were read against the standard template control.

The Anti-nuclear Antibody (ANA) IgG (IIFT) kit was purchased from EUROIMMUN Medizinische Labordiagnositika AG (Lübeck, Germany) and the experiments were conducted according to the manufacturer’s instructions. The human epithelial HEP-2 cell line was used as a substrate. The serum samples were diluted 1:10 with a phosphate-buffer solution. The diluted samples were down-titrated to 1:1000 to determine the end-point positive ANA titer. Nuclear immunofluorescence was visually assessed by two independent microscopists to avoid subjective bias in their conclusions. No difference was observed in the image assessment. Fluorescence signal detection was performed using fluorescent microscopy (Nikon Eclipse 80i; Nikon Corporation, Tokyo, Japan) with ×400 magnification.The image was captured with a Nikon DS-5 Mc digital camera and stored automatically on a computer for subsequent re-inspection. The intensity of each fluorescent image was interpreted as negative (−), weak (1+), moderate (2+), strong (3+) or very strong (4+) and the type as homogenous, speckled, nucleolar or peripheral. To avoid a reduction in the fluorescence signal (i.e., the extinguishing of fluorescence) with time, the determination of the fluorescence model started immediately following the completion of the immune reaction and within 1–2 min per sample with an inspection of ≥5 visual fields. An Anti-dsDNA Antibody IgG kit (IIFT) was used and *Crithidia luciliea* was used as a substrate. The experimental steps were the same as those used with the ANA kit.

### Laboratory parameters

Blood was collected from all patients at the time of diagnosis and tests for the following were performed in our laboratory center: red blood cells (RBCs), white blood cells (WBCs), neutrophils (Ns), lymphocytes (Ls) and platelets (PLTs), erythrocyte sedimentation rate (ESR) and C-reactive protein (CRP). The anti-nuclear antibody (ANA) and anti-dsDNA antibody was measured by an indirect immunofluorescence assay. The anti-Sjögren’s syndrome type A (SSA)60 and anti-SSA52 antibodies were detected by the LIA method.

### Evaluation of SLE activity

The SLE Disease Active Index (SLEDAI) was used to evaluate the disease activity at the time of diagnosis (16).

### Clinical parameters

Cheek erythema, discoid erythema, Raynaud’s phenomenon, arthritis, oral ulcers, photosensitivity, hair loss, serositis (pleuritis or pericarditis), central nervous system damage (psychosis, seizures, organic brain syndrome, transverse myelitis and cranial and peripheral neuropathies), kidney damage (hematuria, proteinuria, casts and nephrotic syndrome) and secondary Sjögren’s syndrome (sSS) were noted.

### Statistical analysis

The statistical analyses were conducted using SPSS 11 software (SPSS, Inc., Chicago, IL, USA). A χ^2^ or Fisher’s test was used for the count data and a Student’s t-test was used for the measurement data. P<0.05 was considered to indicate a statistically significant difference.

## Results

### Sensitivity and specificity of the anti-SSB antibody

Of the 74 SLE patients, 19 cases were anti-SSB antibody-positive, a positive rate of 25.7%. In the 30 controls, 1 case with adult onset Still’s disease was anti-SSB antibody-positive and all the others were negative. The sensitivity of the anti-SSB antibody for diagnosing SLE was 25.7% while the specificity of the anti-SSB antibody was 96.7%.

### Correlation between serum anti-SSB antibody and clinical manifestations

The correlations between anti-SSB antibody positivity and negativity with various clinical manifestations in the 74 SLE patients are shown in Table I.

In the anti-SSB antibody-positive group, the incidences of hair loss, cheek erythema and serositis were significantly higher than in the negative group (P=0.018, 0.023 and 0.035, respectively). In 14 (18.9%) of the 74 SLE patients, the SLE was complicated by sSS, and its incidence in the anti-SSB antibody-positive cases (36.84%) was higher than that in the anti-SSB antibody-negative cases (12.73%; χ^2^=5.353; P=0.021).

### Correlation between serum anti-SSB antibody and experimental parameters

The correlations between anti-SSB antibody positivity and negativity and various experimental parameters are listed in Table II (categorical data) and Table III (measurement data).

The incidences of leukopenia and neutropenia in the anti-SSB antibody-positive group (63.16 and 53.58%, respectively) were significantly higher than those in the anti-SSB antibody-negative group (34.55 and 21.99%; χ^2^=4.749 and 6.418; P= 0.029 and 0.011, respectively). The positive rates of the anti-SSA60, anti-SSA52 and double anti-SSA60 and SSA52 antibodies in the anti-SSB antibody-positive group were higher than those in the anti-SSB antibody-negative group (χ^2^=4.334, 12.052, and 13.069; P=0.037, 0.001 and 0.000 respectively). All cases were ANA positive. There was no significant difference in anti-dsDNA antibodies between the two groups.

The leukocyte and neutrophil counts in the anti-SSB antibody-positive group (4.13±1.57 and 1.20±0.39×10^9^/l, respectively) were lower than those in the anti-SSB antibody negative group (5.23±2.10 and 3.75±2.07×10^9^/l; t=2.086 and 3.506; P=0.040 and 0.015, respectively). The IgG level in the anti-SSB antibody positive group (20.56±4.78 g/l) was higher than that in the anti-SSB antibody negative group (17.46±5.12 g/l; t=2.566; P=0.016). The levels of C3, C4, IgA, ESR, CRP and IgM did not differ between the two groups. The difference in the Systemic Lupus Erythematosus Disease Activity Index (SLEDAI) scores between the anti-SSB antibody-positive (12.00±7.76) and negative groups (10.98±8.19) was not significant (t=0.474, P=0.654).

## Discussion

In 1979, Alspaugh and Maddison identified two new nuclear antigens from the sera of primary Sjögren syndrome patients and named them SSA and SSB (17). Biswas *et al* identified that the SSB antigen (18), the same substance as a cytoplasmic antigen La, namely the SSB/La antigen, was associated with the decreased phagocytic efficiency of neutrophils in patients with SLE. The SSA and SSB antigens are fragments of ribonucleoprotein. The molecular weight of the SSB antigen is 48,000, it is highly conserved in evolution and is involved in regulating RNA polymerase III and mRNA transcription. Amino acid sites 80–100, 220–240 and 300–340 in the SSB molecule have a strong antigenicity (19).

The anti-SSB antibody is one of the most common SLE serum autoantibodies and is produced prior to the onset of SLE (20,21). Previous findings suggest that the anti-SSB antibody is a significant player in the pathogenesis of SLE and that it is also relatively stable in the course of SLE. This is supported by the fact that the anti-SSB antibody is independent of the SLEDAI score in the present study. The positive rate of the anti-SSB antibody in the SLE patients was 25.7%. The difference between these findings and those of previous studies may be due to variations in detection methods. The present study has shown that, when excluding rheumatoid arthritis (RA) and primary Sjögren’s syndrome (pSS), the specificity of anti-SSB antibody for diagnosis of SLE is as high as 96.7%. Occasionally, it is not possible to establish a diagnosis immediately and, in such cases, the patients should be closely followed-up in order to avoid a misdiagnosis and delay in treatment.

A previous study has also shown that the anti-SSB antibody is involved in the formation of an idiotype-anti-idiotype network in the pathogenesis of SLE, which has stimulated increased interest in the topic (22). Routsias *et al* identified that the complementary peptide 289–308 amino acids of the SSB antigen share similar or identical amino acid sequences with *Mycobacterium tuberculosis*(23), *Helicobacter pylori*, *Streptococcus*, *Escherichia coli* and malaria parasites, suggesting that the anti-SSB antibody may simulate these biological agents and cause disease. In the present study, the anti-SSB antibody was identified to be associated with cheek erythema, alopecia and serositis. The mechanism behind this is unknown and may be related to the deposition or formation of a local antigen-antibody complex, which causes local vascular inflammation, increased vascular permeability, or microcirculatory disturbance.

In the present study, leukopenia and neutropenia were identified to correlate with the SSB antibody. It has been recognized that neutropenia is a consequence of anti-polymorphonuclear neutrophil (PMN) antibodies, but the nature of the anti-PMN antibody is not clear. Previously, Hsieh *et al* confirmed for the first time that the anti-SSB antibody is an anti-PMN antibody (12). Studies suggest that the anti-SSB antibody is not only able to bind to the surface of PMNs but also enter PMNs to induce antibody-dependent cellular cytotoxicity or activation-induced cell death (AICD), which may cause a decline in the phagocytosis and apoptosis of the PMNs. Neutrophils comprise the majority of the leukocytes in the peripheral blood. Leukopenia is also related to the anti-SSB antibody. In total, ∼8–30% patients have SLE complicated with sSS due to unknown causes. The present study identified that the incidence of sSS varies between the anti-SSB antibody-positive and negative groups, strongly suggesting that the anti-SSB antibody is associated with sSS. The IgG immunoglobulin levels were also significantly increased, suggesting a specific secretion of the IgG anti-SSB antibody in activated B cell clones in patients with SLE.

SSB and SSA60 molecules are different ribonucleoprotein fragments. SSA52 has not been classified as a fragment of ribonucleoprotein, but it is able to transiently bind with SSA60 (24). In the present study, the anti-SSB antibody was frequently present with the anti-SSA60 or anti-SSA52 antibodies. The specific mechanisms for this correlation are unknown and deserve further study.

In conclusion, anti-SSB antibodies are associated with cheek erythema, alopecia, serositis, sSS, leukocytopenia, elevated IgG levels and positivity for anti-SSA60 or anti-SSA52 antibodies. Anti-SSB may be used as a criterion for the early diagnosis of SLE. As the present study is a clinical observation, further molecular biological study is necessary to confirm the pathogenic mechanism of SLE.

## Figures and Tables

**Table I:** Correlation of anti-SSB antibody positivity and negativity with various clinical manifestations.

	Anti-SSB antibody-positive (n=19)		Anti-SSB antibody-negative (n=55)		c^2^	P-value
	
Clinical parameters	Cases	Incidence (%)	Cases	Incidence (%)		
Raynaud’s phenomenon	3	15.79	3	5.45		0.172^*^
Arthritis	11	57.89	35	63.64	0.198	0.656
Cheek erythema	13	68.42	21	38.18	5.199	0.023
Discoid erythema	1	0.52	0	0.00		0.257^*^
Oral ulcers	2	10.52	12	21.82	1.174	0.279
Photoallergy	5	26.32	12	21.82	0.161	0.688
Hair loss	6	31.58	5	9.09	5.643	0.018
Serositis	6	31.58	6	10.91	4.441	0.035
Central nervous system damage	1	0.52	4	7.27		1.000^*^
Kidney damage	12	63.16	22	40.00	3.049	0.081
sSS	7	36.84	7	12.73	5.353	0.021

* Fisher’s Exact Test. SSB, Sjögren’s syndrome type B; sSS, secondary Sjögren’s syndrome.

**Table II:** Correlation of anti-SSB antibody positivity and negativity with various experimental parameters (categorical data).

	Anti-SSB antibody-positive (n=19)		Anti-SSB antibody-negative (n=55)		χ^2^	P-value
	
Experimental parameters	Cases	Incidence (%)	Cases	Incidence (%)		
Leukopenia	12	63.16	19	34.55	4.749	0.029
Neutropenia	10	53.58	12	21.99	6.418	0.011
Low C3	3	37.50	19	65.51		0.228
Low C4	1	12.50	0	0.00		0.216
High IgA	2	25.00	4	13.33		0.587
High IgG	7	87.50	12	40.00		0.042
High IgM	1	12.50	2	6.67		0.519
SSA60	17	89.47	33	60.00	4.334	0.037
SSA52	16	84.21	19	34.55	12.052	0.001
SSA60 + SSA52	16	84.21	18	32.73	13.069	0.000

SSB, Sjögren’s syndrome type B; SSA, Sjögren’s syndrome type A; Ig, immunoglobulin; C3, complement component 3; C4, complement component 4.

**Table III:** Correlation of anti-SSB antibody positivity and negativity with various experimental parameters (measurement data).

	Anti-SSB antibody-positive (n=19)		Anti-SSB antibody-negative (n=55)		t	P-value
	
Experimental parameters	Mean	SD	Mean	SD		
SLEDAI	12.00	7.76	10.98	8.19	0.474	0.654
Leukocytes (10^9^/l)	4.13	1.57	5.23	2.10	2.086	0.040
Neutrophils (10^9^/l)	1.20	0.39	3.75	2.07	3.606	0.015
IgG (g/l)	20.56	4.78	17.46	5.12	2.566	0.016
IgM (g/l)	1.81	3.21	1.58	3.55	0.249	0.778
IgA (g/l)	3.14	3.25	3.50	3.04	0.437	0.645
C3	0.56	0.33	0.75	0.45	1.687	0.078
C4	0.17	0.16	0.19	0.07	0.749	0.447
ESR (mm/1st h)	45.55	14.36	40.38	13.55	1.412	0.149
CRP (mg/l)	13.55	5.45	10.77	6.21	1.732	0.072

SSB, Sjögren’s syndrome type B; SLEDAI, Systemic Lupus Erythematosus Disease Activity Index; Ig, immunoglobulin; ESR, erythrocyte sedimentation rate; CRP, C-reactive protein; C3, complement component 3; C4, complement component 4.
